# Applications of artificial intelligence in musculoskeletal ultrasound: narrative review

**DOI:** 10.3389/fmed.2023.1286085

**Published:** 2023-11-21

**Authors:** Stefan Cristian Dinescu, Doru Stoica, Cristina Elena Bita, Andreea-Iulia Nicoara, Mihaela Cirstei, Maria-Alexandra Staiculesc, Florentin Vreju

**Affiliations:** ^1^Department of Rheumatology, University of Medicine and Pharmacy of Craiova, Craiova, Romania; ^2^Physical Education and Sport Department, Motor Activities Theory and Methodology, Craiova University, Craiova, Romania; ^3^University of Medicine and Pharmacy Craiova, Craiova, Romania

**Keywords:** artificial intelligence, deep learning, machine learning, ultrasonography, musculoskeletal system

## Abstract

Ultrasonography (US) has become a valuable imaging tool for the examination of the musculoskeletal system. It provides important diagnostic information and it can also be very useful in the assessment of disease activity and treatment response. US has gained widespread use in rheumatology practice because it provides real time and dynamic assessment, although it is dependent on the examiner’s experience. The implementation of artificial intelligence (AI) techniques in the process of image recognition and interpretation has the potential to overcome certain limitations related to physician-dependent assessment, such as the variability in image acquisition. Multiple studies in the field of AI have explored how integrated machine learning algorithms could automate specific tissue recognition, diagnosis of joint and muscle pathology, and even grading of synovitis which is essential for monitoring disease activity. AI-based techniques applied in musculoskeletal US imaging focus on automated segmentation, image enhancement, detection and classification. AI-based US imaging can thus improve accuracy, time efficiency and offer a framework for standardization between different examinations. This paper will offer an overview of current research in the field of AI-based ultrasonography of the musculoskeletal system with focus on the applications of machine learning techniques in the examination of joints, muscles and peripheral nerves, which could potentially improve the performance of everyday clinical practice.

## 1 Introduction

The use of artificial intelligence (AI) technologies in medical imaging is an intensely studied topic in today’s research (1). AI-based methods are used by researchers in order to recognize complex patterns, quantify and interpret features of imaging data, which have potential roles in both diagnosis and therapy monitoring. The need for standardization of imaging assessments and increase in computational power has led to exponential growth of AI-based research in medical imaging. Machine learning (ML) is a subset of AI which uses pattern recognition, learns from data, makes predictions and enables decision making on future data (2). Deep learning (DL), a subclass of ML, uses layered-structure algorithms, such as artificial neural network (ANN) and convolutional neural networks (CNN) to process significant amounts of data (3). In particular, CNN have allowed for a remarkable progress in the process of image recognition (2, 4). Ultrasound (US) has gained widespread use in rheumatology practice, although operator-dependent which means that it is also prone to subjective interpretive errors (4). There are multiple AI-based techniques which can improve the accuracy of US assessment. These include: automated image acquisition (5, 6), image interpretation, anatomical landmarking (7, 8), measurement and quantification, probe positioning guidance (9), elastography interpretation (10) and fusion with other imaging modalities. AI applications could improve US assessment of patients with inflammatory rheumatic diseases by means of detecting and quantifying of inflammatory lesions or of structural changes of cartilage and bone. Performing local procedures can also benefit from accurate anatomical landmarking through AI-assisted methods (7, 11). This narrative review offers insight into current machine learning techniques applied in ultrasound imaging and focus on the specific applications for the examination of joints, muscles and peripheral nerves, which could potentially improve the performance of everyday clinical practice.

## 2 AI-based techniques for ultrasound imaging

AI is broadly defined as a computer system that performs tasks which would typically rely on human intelligence. ML is a subfield of AI which involves algorithms that learn and make decisions from input data. Computer aided diagnosis (CAD) systems have been introduced in clinical practice for over two decades. Initial applications of CAD in radiology have proven successful in automatic tumor detection and monitoring (1). Research on AI-assisted medical imaging has witnessed a rapid growth in recent years (12). Ultrasound is a highly operator-dependent imaging method which relies heavily on the examiners experience and also the ultrasound machine available in each clinical setting. These limitations increase the variability in image acquisition and interpretation. CAD systems have the potential to overcome some of these drawbacks by improving accuracy and consistency of US assessment and providing the examiner a second opinion during image interpretation (13).

The first CAD systems were based on ML techniques. Multiple ML model types have been studied for their performance in the assessment of imaging features. ML models are generally classified in supervised, unsupervised methods. Supervised learning is the most common type and it involves the preparation of a training data set. Thus, a golden standard is defined, the so called ground truth, through labeling of data content by an expert. ML algorithms used in imaging research include k-nearest neighbor, decision trees, random forest and support vector machine (14, 15).

One fundamental aspect of ML techniques is that they require some level of human inference during region of interest (ROI) selection and feature extraction. Thus, input provided to the ML algorithms relies heavily on the examiners knowledge. This has led to the development of more complex DL models which bypasses this manual feature engineering (13). DL pipelines contain multiple hidden neural network layers which have been essential for the development of end-to-end learning techniques. Training in DL models usually follows a supervised approach which involves three data set categories: training, validation and testing data which evaluates the generalizability of the developed model on new data (16).

CNNs have been widely used in image processing applications. CNNs are biologically-inspired neural networks which contain a series of hidden layers that respond to specific features. CNNs are composed of three layer domains: convolutional layer, pooling layer and fully connected layer. The convolutional layer contains several filters that generate a two-dimensional activation map. The image undergoes several convolutions which are processed to extract high-level features. The pooling layer reduces the spatial dimensions through down-sampling and extracts the optimized output. Finally, a fully-connected layer acts as a classifier and assigns a relevant category depending on the purpose of the model (17).

ML models developed for musculoskeletal ultrasound (MSUS) usually focus on segmentation, diagnosis and classification. There are multiple clinical settings in which application of ML models can aid MSUS. These include the assessment of synovial tissue (15, 18), tendon (19, 20), cartilage (21) and nerve identification (11, 22, 23). When examining these structures, a ML algorithm can perform either recognition or a diagnostic task. Localization of the specific area of interest can be followed by a segmentation process which aims to highlight a precise contour of an anatomical structure (24). Depending on the model’s application, the diagnostic output can be binary, which decides if the image meets a diagnosis or not, or multiclass if the model must grade the pathologic findings (24). Importantly, the classification performance of every CAD system relies on the quality of the raw US images. Thus, image pre-processing is an essential step prior to the input in a ML algorithm. Image enhancement has also become a domain for the implementation of DL models with aim to overcome the limitations of conventional beanforming techniques (17).

## 3 Joint and tendon assessment

US is a very useful imaging tool for the evaluation of patients with inflammatory joint pathology. It offers many advantages for clinical practice such as the ability to evaluate a joint in real-time, scan for multiple sites and direct correlation of clinical data with imaging findings. The added value of US has been proven for a wide range of rheumatic diseases. Well established guidelines published by the European Alliance of Associations for Rheumatology (EULAR) recommend the use of US in the assessment of patients with rheumatoid arthritis (25, 26), spondyloarthritis (27), gout (28) and calcium pyrophosphate deposition disease (CPPD) (29).

### 3.1 Synovitis

One of the main benefits of using ultrasound in the assessment of patients with joint pain is the ability to confirm if synovitis is present and establish its severity. In addition to its diagnostic use, US can guide physicians in order to perform more accurate joint aspirations, injections and synovial biopsies (30).

The EULAR/OMERACT scoring system published by the Outcome Measures in Rheumatology (OMERACT) US Working Group has been developed to grade synovitis by greyscale and Doppler mode in a standardized manner (31). In daily practice, scanning multiple joints coupled with semiquantitative assessment of synovitis can become a time consuming task which is subject to increased interreader variability depending on the sonographers experience and quality of US machine (32). CAD systems have been developed and tested in studies which have proven their potential for both detection and grading of synovitis (15, 33–39). Most DL models developed to quantify synovitis integrate a CNN-based framework trained on previously scored images labeled for ground truth (39).

A common algorithm pipeline for classification models designed to detect synovitis includes skin border and bone line detection, followed by synovial region segmentation. Additionally, attention maps are integrated in the model in order to further highlight areas of interest (13). Radlak et al. (35) applied an automatic algorithm using seeded region growing for synovial segmentation with different noise filtering methods. The proposed model offered high-quality segmentation output with many images showing overlap between automated traced areas of synovitis and the manually delineated regions. One of the first classification systems for grading synovial proliferation was described by Mielnik et al. (15) in 2018. The model was tested on 140 US images of metacarpophalangeal and proximal interphalangeal joints obtained from patients with chronic arthritis. They reported a moderate agreement between algorithm and ground truth and also between algorithm and human examiners which were involved in the validation process. More recent DL models report high accuracy for synovitis detection (34, 36, 37). Tang et al. (37) developed an algorithm for classifying synovitis in rheumatoid arthritis patients using deep CNNs and reported accuracies exceeding 90% for both binary classification and grading on a 4-point scale. Most CAD systems developed for synovitis detection and scoring are trained only on gray-scale US images. Nevertheless, some models, like the ones described by Andersen et al. (38) and He et al. (33), also integrate Doppler mode in order to grade synovitis based on the OMERACT-EULAR synovitis scoring system (31).

### 3.2 Tendon pathology

Computer assisted tendon segmentation models have been studied for their clinical applicability. Alzyadat et al. (40) provided promising results using a CNN-based framework for Achilles tendon automatic segmentation. Two studies from 2020 have proven the feasibility of implementing CAD systems for automated recognition of supraspinatus (SSP) tendinopathy (4, 20). In the study by Jahanifar et al. (20), a CNN-based model was trained and validated for classification of SSP tendinopathy with 91% accuracy. Chin et al. (4) developed a DL recognition model to differentiate US images based on the presence or absence of SSP calcifications with 91% accuracy. Another practical application of tendon segmentation is the assessment of finger flexors which are involved in the occurrence of trigger finger. Kuok et al. (19) developed a deep CNN model for identifying tendon and synovial sheath which could be integrated in US-guided systems that assist trigger finger surgery.

## 4 Cartilage pathology

Cartilage damage is one of the main features of osteoarthritis (OA), a common degenerative disorder among the elderly population. Compared to MRI, US allows for a faster and more accessible imaging approach to cartilage description. Morphometric features of cartilage examined through US include changes in echogenity, crystal deposits, surface irregularities and thickness measurement. AI models developed for cartilage examination focus on image enhancement, automated segmentation and thickness measurements (21, 41–45). Hossain et al. (46) describes a histogram equalization method that achieves a comprehensive contrast enhancement of the knee cartilage which provides better quality images and can be later integrated in an automated detection system. Performance of AI-based knee cartilage segmentation models measured through dice similarity coefficient upon validation with manually delineated images has shown promising results (21, 41–45). These techniques have important clinical implications in early detection of knee osteoarthritis. Furthermore, automated segmentation techniques could minimize the risk of surgery-related cartilage damage during robotic knee arthroscopy. Antico et al. (21) implemented a U-Net framework based method for cartilage segmentation in dynamic, volumetric US images, designed to help avoid contact between healthy tissue and surgical instruments. This algorithm provided good accuracy for femoral cartilage localization, which supports its potential application in robotic knee arthroscopy. Quantifying femoral articular cartilage can be achieved through 3D US assessment and this has been validated with the standard MRI approach (45). Toit et al. (44) developed a DL model for 3D femoral cartilage reconstruction. They reported no significant difference in automated cartilage volume estimation compared to manual 3D segmentation. Apart from the knee joint, measurements of cartilage thickness can be performed at other joint sites, for example in order to assess rheumatoid arthritis-related cartilage damage at the level of the hands. In a 2022 study, Fiorentino et al. (47) applied a CNN framework designed for automated cartilage thickness measurement of the metacarpal head. This proposed DL model performed comparable to the intra-observer variability.

## 5 Skeletal muscle disorders

US is an essential and easy to perform imaging method for rapid detection of muscle injuries and has additional diagnostic implications for muscle disorders such as muscle dystrophy and idiopathic inflammatory myopathies (IIM). US can help visualize the muscle structure and assess the motor function in real-time. Changes in echogenity and thickness are some of the main features of IIM and muscular dystrophies. In IIM, echogenity increases and is more pronounced in the chronic phase when it is also accompanied by a reduced muscle thickness. US features predictive of muscle dystrophy include a significant increase in echogenity with “ground glass appearance,” with attenuation in deeper layers and loss of normal muscle architecture (48). Some important prerequisites for the analysis of skeletal muscle US images include boundary identification and muscle size measurement. These tasks can be time consuming and could thus benefit from the use of automated segmentation and quantification methods (49–51). Furthermore, AI-based analysis and classification systems of muscle texture features have important clinical applications in the diagnosis and monitoring of skeletal muscle pathologies (52).

Some of the first automated muscle segmentation models showed good recognition capabilities in healthy individuals. The MUSA algorithm developed by Caresio et al. (53) applied the gradient-based filter to measure muscle thickness by delineating the superficial and deep aponeurosis of gastrocnemius muscle in longitudinal sections. The TRAMA algorithm proposed by Salvi et al. (54) was one of the first fully automatic models for the extraction of muscle cross-sectional area (CSA) using transverse section images of rectus femoris and gastrocnemius muscle. More recent developments rely on CNN-based models for muscle segmentation with improved recognition and CSA extraction output (49).

DL models designed for the analysis of muscle structure have been researched in order to develop CAD systems for IIM or muscular dystrophies. Burlina et al. (55) proposed a semiautomatic classification method which achieved 86% accuracy for distinguishing US images of IIM from healthy muscle. Upon measuring its performance in differentiating between IIM subtypes the model obtained only 68% accuracy. Ucar et al. (56) tested binary and multiple classification scenarios for IIM. Importantly, this DL model achieved high diagnostic performance for each scenario and could accurately differentiate between inclusion-body myositis and polymyositis or dermatomyositis.

AI-based classification systems for muscular dystrophies are designed to analyze muscle morphology, as well as ambulatory function through measurement of fascial length and pennation angle. Several ML and DL models have been developed for distinguishing US images of muscular dystrophies from healthy muscle tissue with satisfying performance. Srivastava et al. (57) developed a ML technique based on support vector machine algorithm for quantitative US evaluation combined with electrical impedance myography in order to distinguish between several subtypes of muscular dystrophies. Cunningham et al. (58) applied a DL model based on CNN architecture together with synchronous electromyography (EMG) examination of the calf muscles during active contraction and passive joint rotation. The imaging features extracted by the AI algorithm could predict specific EMG patterns and the state of muscle activity. These findings support the use of AI-based US as a non-invasive alternative to EMG in the assessment of muscular dystrophy.

## 6 Peripheral nerve assessment

Main applications of peripheral nerve US imaging consist in pathology diagnosis through structure analysis and guidance of local procedures such as nerve blocks. Sometimes the anatomical positioning and small CSA make accurate detection of peripheral nerves challenging. AI-based techniques could optimize the US examination of peripheral nerves through image enhancement, real-time segmentation and quantitative measurements (13).

Automated segmentation of peripheral nerves reduces the time-consuming task of nerve delineation and manual measurements. ML-based algorithms designed for US nerve segmentation involve a process of despeckle filtering, followed by ROI detection and classification of nerve region (13, 14). DL-based segmentation methods which integrate U-Net architecture have been developed in order to reduce the degree of human intervention and bypass some intermediate stages of ML pipelines (13, 59). Smistad et al. (59) applied a CNN-based model to detect musculocutaneous, median, ulnar, and radial nerves, while also testing several augmentation methods. Models based on CNN framework have also been developed for brachial plexus (13) and femoral nerve segmentation (23).

One important application of using automated AI-based methods for nerve segmentation and identification of regional anatomical landmarks is the real-time guidance of peripheral nerve block procedures. This is particularly important for training and gaining clinical experience (23). Studies by Berggreen et al. (23) and Huang et al. (11) report good performance of DL models based on U-Net framework applied for recognition of femoral nerve which could assist regional anesthesia. Gungor et al. (7) studied the accuracy of an AI-based real-time identification tool of anatomical landmarks to assist infraclavicular, supraclavicular, interscalene and transverse abdominis plane blocks during US-guided procedures. Bowness et al. (8) studied a CNN-based model based on U-Net framework to segment the input of US videos. The model provided highlighting of segmented anatomical structures through color overlay and achieved a very high accuracy in recognizing specific anatomical structures. These promising results support the potential of AI-based systems in assisting US-guided regional anesthesia.

## 7 Conclusion

The adoption of artificial intelligence techniques has revolutionized the field of medical imaging. Ultrasonography is known to be an operator-dependent imaging modality and this poses certain limitations that can be overcome by the integration of AI-based tools which could enhance the quality of scans and standardize the process of image acquisition. This review highlights the current research in the field of AI-based musculoskeletal ultrasonography which has proven its potential use in various clinical settings. Further studies are still required to prove the applicability of AI models in the detection of tenosynovitis or bone pathologies (e.g., erosion) in inflammatory joint diseases. Accurate detection and monitoring of rheumatic and musculoskeletal diseases rely on high quality input from imaging assessment. AI techniques developed for specific imaging tasks can thus become an essential supplementary tool to clinical reasoning.

## Author contributions

SD: Conceptualization, Writing – original draft, Writing – review & editing. DS: Conceptualization, Writing – review & editing. CB: Writing – original draft. A-IN: Writing – original draft. MC: Writing – original draft. M-AS: Writing – review & editing. FV: Conceptualization, Supervision, Writing – original draft, Writing – review & editing.
